# Supplementary material for: Optoacoustic brain stimulation at submillimeter spatial precision
Source: Nat Commun. 2020 Feb 14;11:881. doi: 10.1038/s41467-020-14706-1 (PMC7021819; doi:10.1038/s41467-020-14706-1)
Supplement: Supplementary file 2 — Supplementary Information [file 41467_2020_14706_MOESM2_ESM.docx]

Supporting Information

**Optoacoustic Brain Stimulation at Submillimeter Spatial Precision**

Ying Jiang et al.


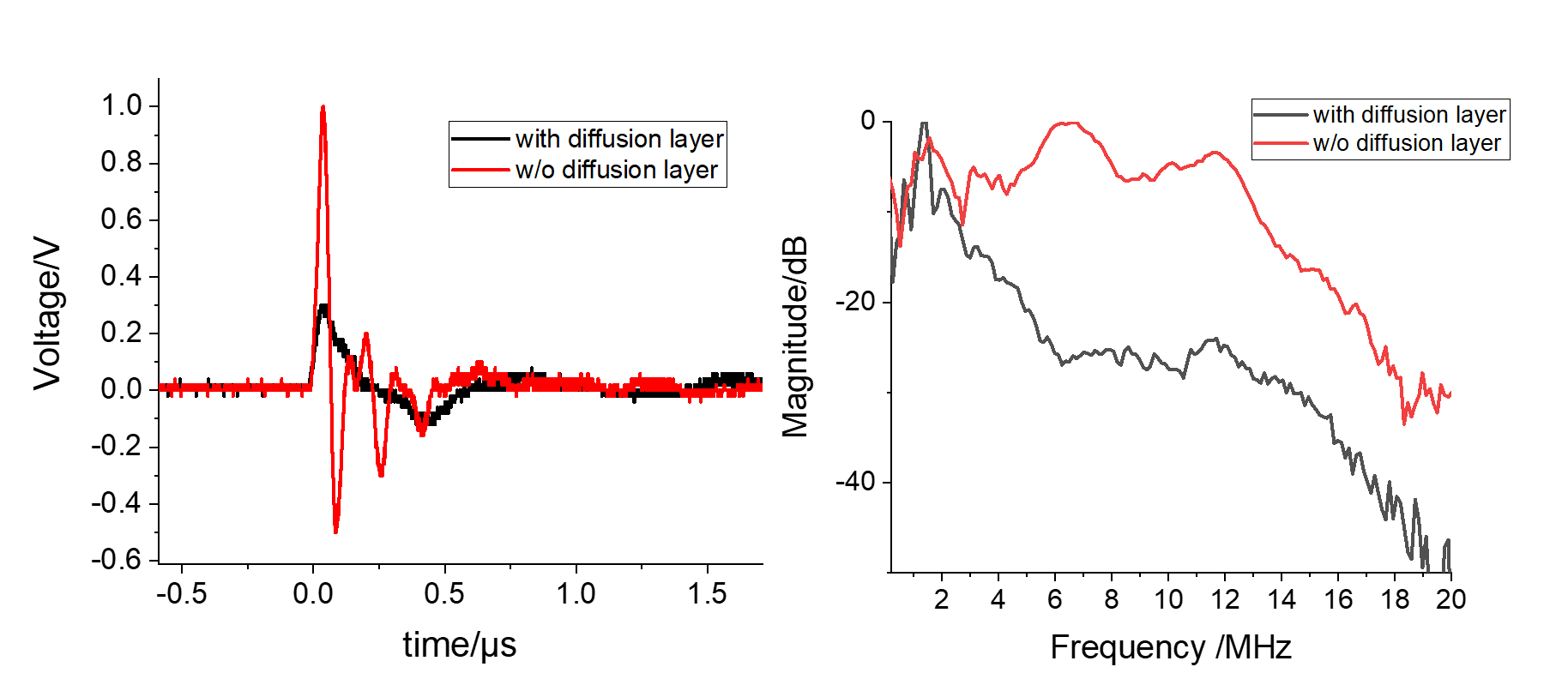


**Supplementary Figure 1.** The diffusion layer at the fiber tip shifts the optoacoustic wave to lower frequency range.

**Supplementary Figure 2.** Angular distribution of light intensity after diffusion layer.


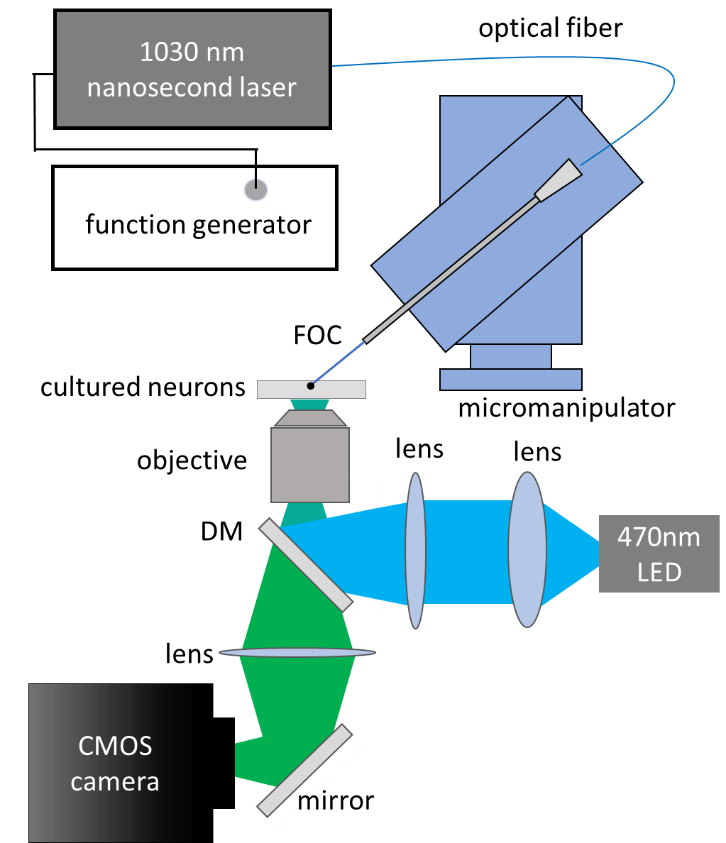


**Supplementary Figure 3.** FOC integrated with an inverted wide field fluorescence microscope. DM: dichroic mirror


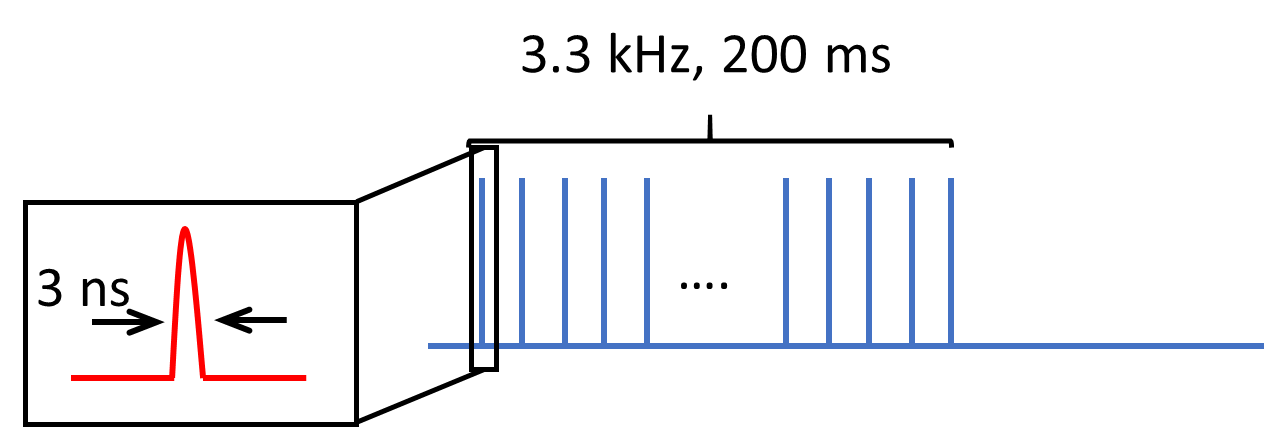


**Supplementary Figure 4.** Illustration of a laser pulse train for 200 ms stimulation.


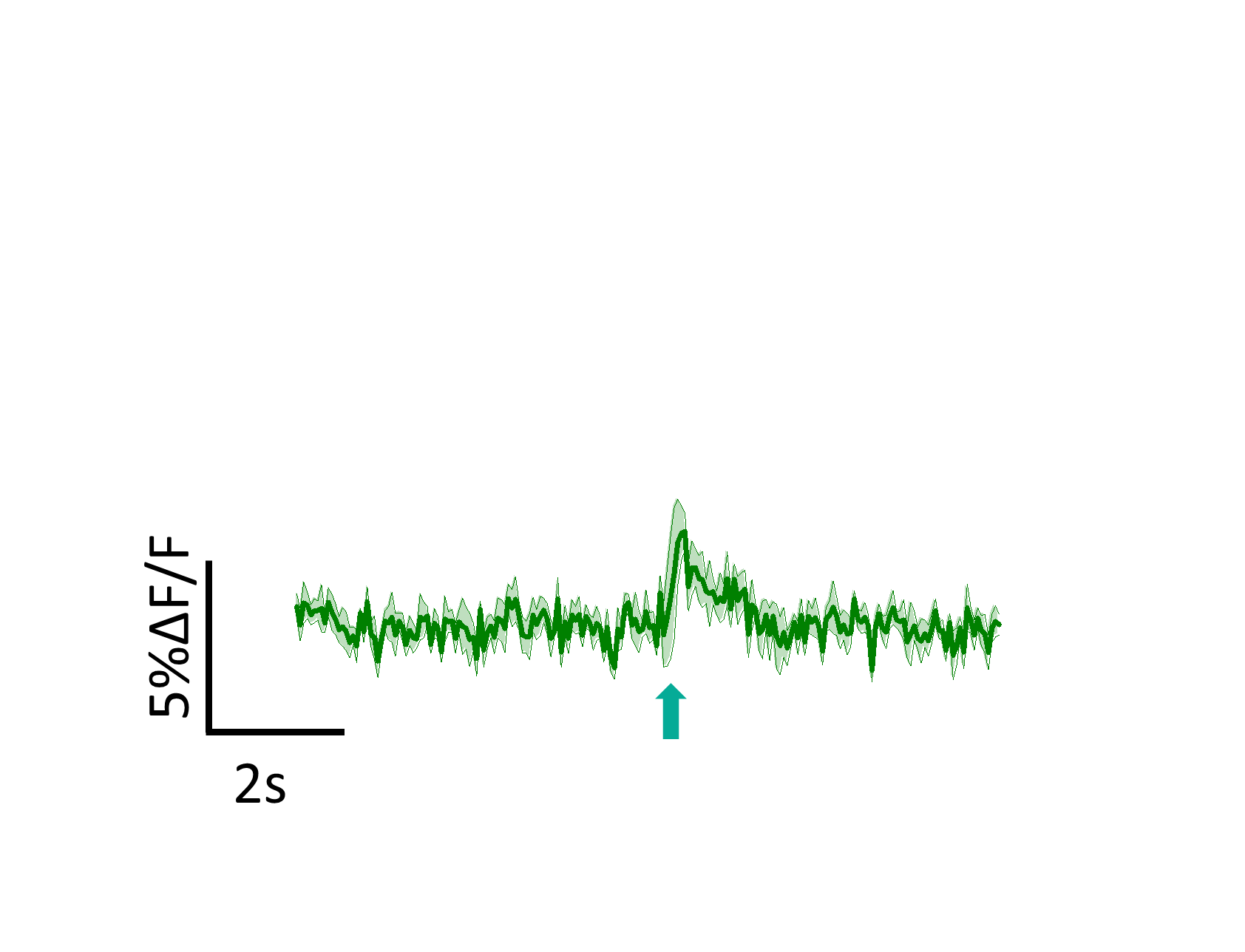


**Supplementary Figure 5.** The 15 μM Calcium chelator BAPTA significantly blocks neural response to 200-ms FOC stimulation (n = 12). Shaded area: ± SD. Green arrow: stimulation onset.

**
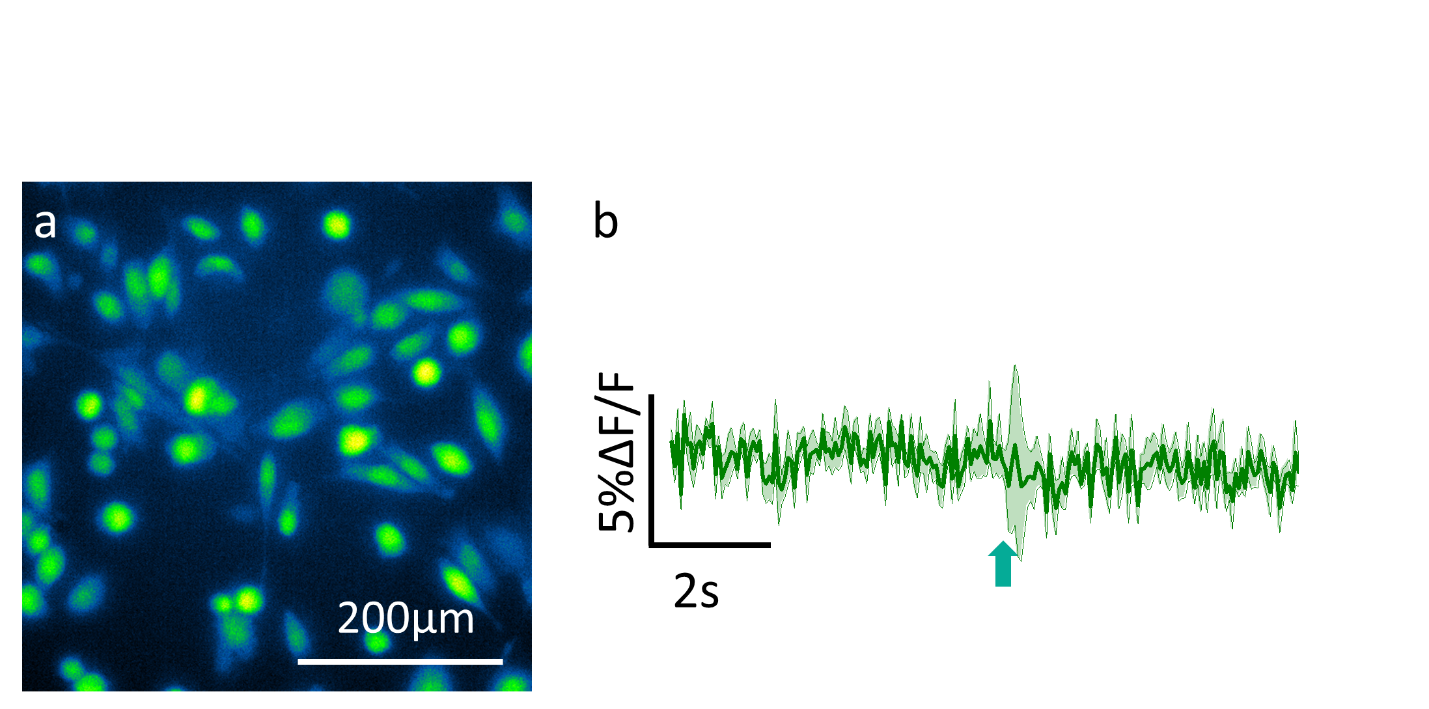
**

**Supplementary Figure 6.** PC3 cells show no response to FOC stimulation. **a**, Oregon Green labelled PC3 cells. **b**, Calcium response to a 200-ms FOC stimulation indicated by the green arrow (n = 52). Shaded area: ± SD. Green arrow: stimulation onset.

**
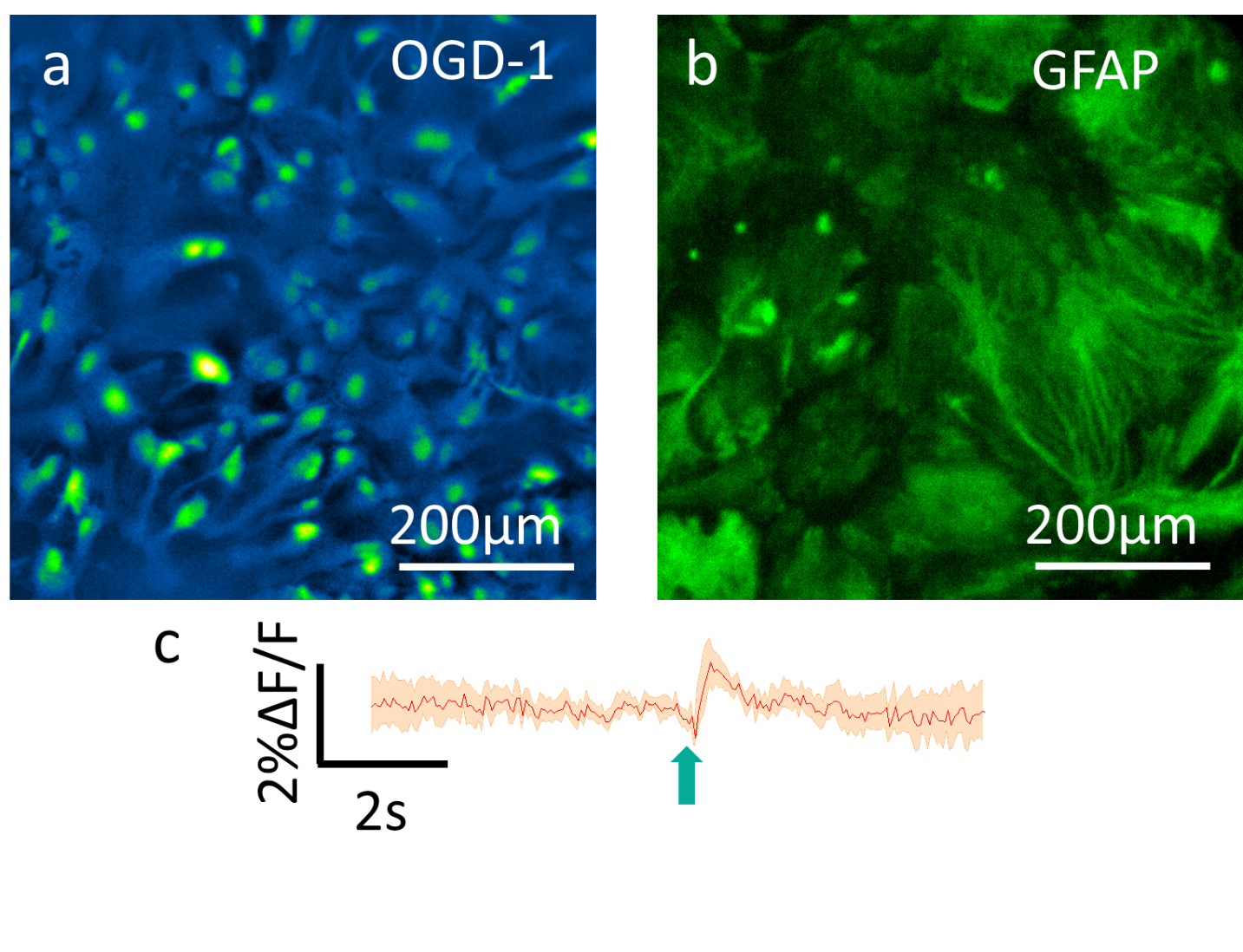
**

**Supplementary Figure 7.** Glial cells show significantly smaller response to 200 ms FOC stimulation. **a**, OGD-1 loaded glial cell culture. **b**, Glial cell culture stained with GFAP. **c**, Glial response to 200-ms FOC stimulation (n = 82). Shaded area: ± SD, Green arrow: stimulation onset.

**
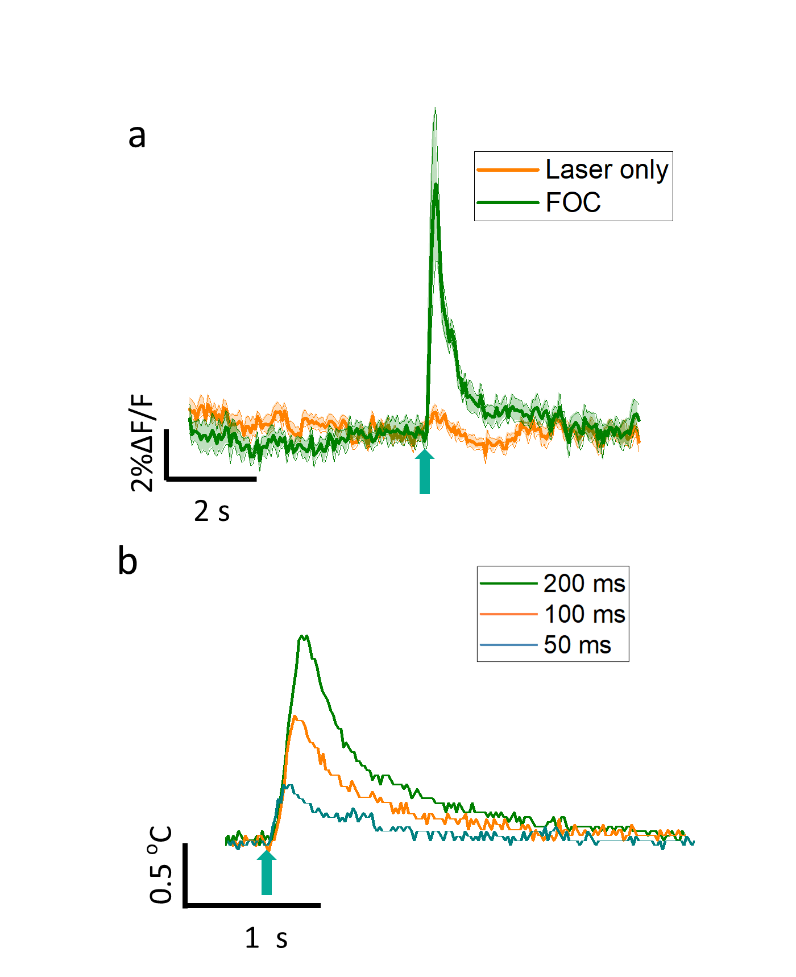
**

**Supplementary Figure 8. FOC-mediated neural activation is not induced by laser or heat. a,** Average trace of neuronal Calcium trace in response to lase stimulation and FOC stimulation. Shaded area: ± SD. **b,** Surface temperature dynamics of FOC tip during laser excitation. Green arrow: stimulation onset.

**
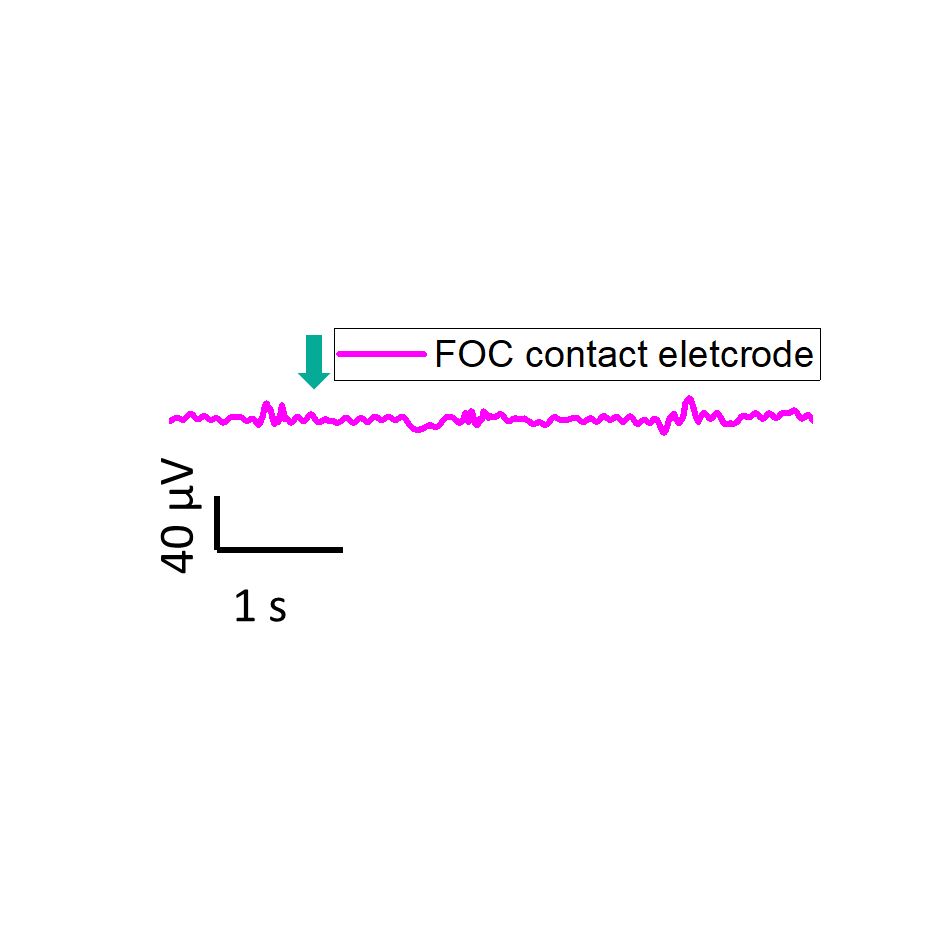
**

**Supplementary Figure 9.** No electronic artifact induced by FOC on the recording electrode. Green arrow: stimulation onset.

**
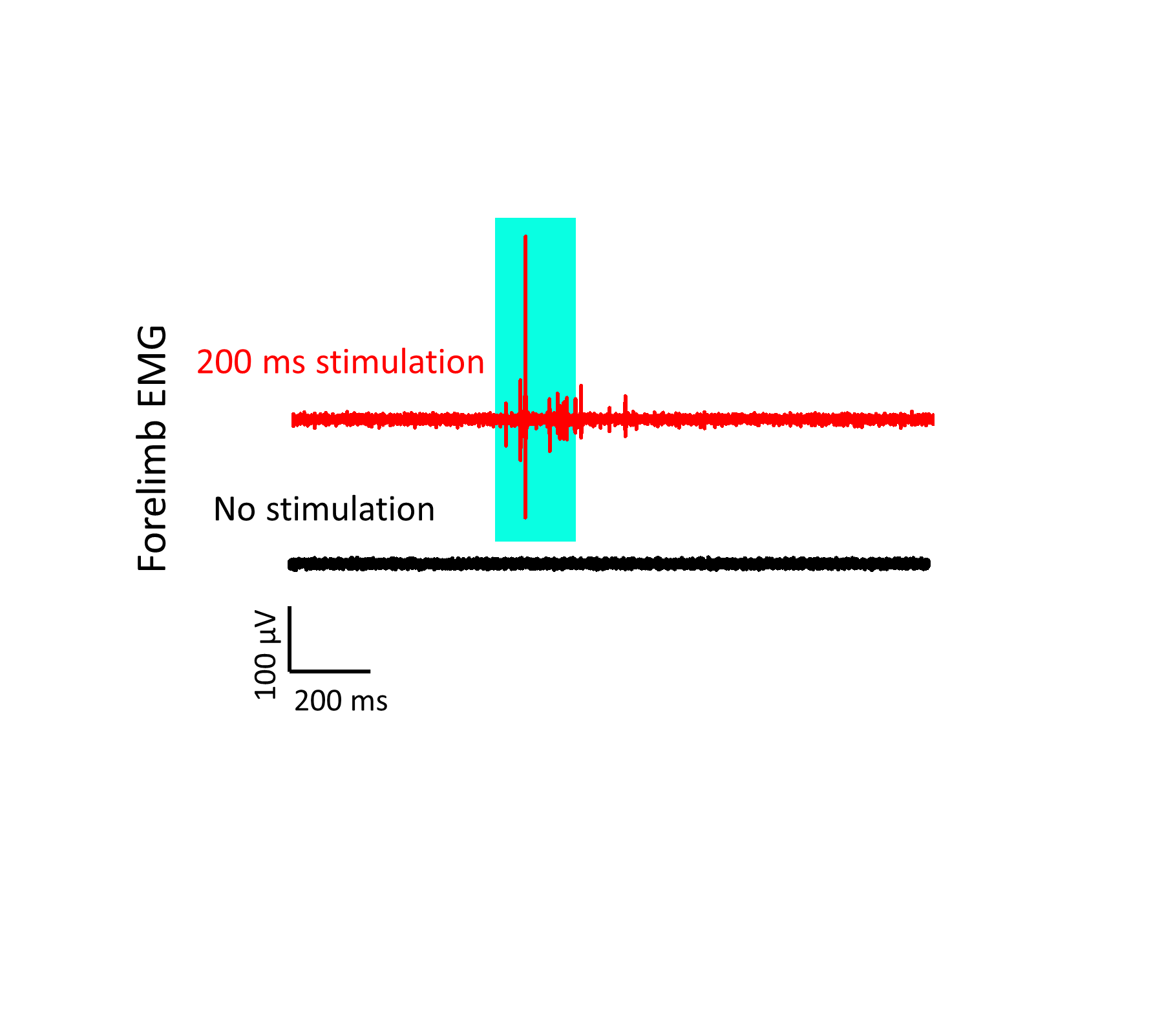
**

**Supplementary Figure 10.** FOC stimulation of the motor cortex induces an EMG response recorded from contralateral forelimb. Blue box: stimulation duration.

**
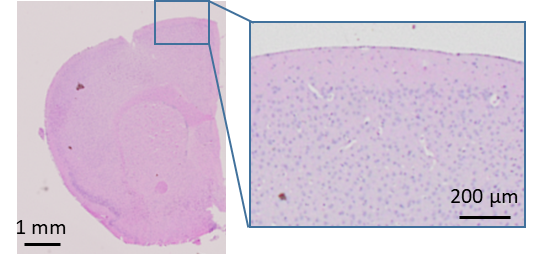
**

**Supplementary Figure 11.** H&E staining of a coronal brain section. Insert shows zoomed in image of motor cortex surface after FOC stimulation.
