# Supplementary material for: Optoacoustic brain stimulation at submillimeter spatial precision
Source: Nat Commun. 2020 Feb 14;11:881. doi: 10.1038/s41467-020-14706-1 (PMC7021819; doi:10.1038/s41467-020-14706-1)
Supplement: Supplementary file 4 — Description of Additional Supplementary Files [file 41467_2020_14706_MOESM4_ESM.pdf]

Title: Supplementary Movie 1
Description: Repeated FOC stimulation of primary cortical neurons. Our point-to-point responses to the reviewers and response on the format check are attached separately.

■Photonics Center ■8 Saint Mary's Street ■Boston, MA 02215
■(617) 353-1276 ■Fax: ■jxcheng@bu.edu
